# Ability of matrix metalloproteinase‐8 biosensor, IFMA, and ELISA immunoassays to differentiate between periodontal health, gingivitis, and periodontitis

**DOI:** 10.1111/jre.12985

**Published:** 2022-03-19

**Authors:** Kehinde Adesola Umeizudike, Hanna Lähteenmäki, Ismo T Räisänen, John J Taylor, Philip M Preshaw, Susan M Bissett, Taina Tervahartiala, Solomon O Nwhator, Pirjo Pärnänen, Timo Sorsa

**Affiliations:** ^1^ Department of Preventive Dentistry Faculty of Dental Sciences College of Medicine, University of Lagos Idi‐Araba Lagos Nigeria; ^2^ Department of Oral and Maxillofacial Diseases University of Helsinki Helsinki University Hospital Helsinki Finland; ^3^ School of Dental Sciences and Translational and Clinical Research Institute Newcastle University Newcastle upon Tyne UK; ^4^ School of Dentistry University of Dundee Dundee UK; ^5^ Department of Preventive and Community Dentistry Faculty of Dentistry College of Health Sciences Obafemi Awolowo University Ile‐Ife Nigeria; ^6^ Division of Periodontology Department of Dental Medicine Karolinska Institutet Stockholm Sweden

**Keywords:** active/total matrix metalloproteinase‐8; saliva, biosensor, periodontal disease

## Abstract

**Objective:**

The aim of this study was to determine the diagnostic utility of an MMP‐8 biosensor assay in differentiating periodontal health from gingivitis and periodontitis and compare it with an established time‐resolved immunofluorescence assay (IFMA) and enzyme‐linked immunosorbent assay (ELISA).

**Background:**

Currently available antibody‐based assays display a wide variability in their ability to accurately measure matrix metalloproteinase‐8 (MMP‐8) levels in saliva.

**Methods:**

Salivary MMP‐8 levels were analyzed in 189 systemically healthy participants using an antibody‐based biosensor prototype that operates using a surface acoustic wave technology and compared with IFMA and ELISA antibody assays. Participants were categorized into 3 groups: periodontal health (59), gingivitis (63), and periodontitis (67). A sub‐population of participants (*n* = 20) with periodontitis received periodontal treatment and were monitored for 6 months.

**Results:**

All the assays demonstrated significantly higher salivary MMP‐8 concentrations in participants with periodontitis versus gingivitis, periodontitis versus health, and gingivitis versus health (all *p* < .05). The biosensor data demonstrated significant correlations with IFMA (*r* = .354, *p* < .001) and ELISA (*r* = .681, *p* < .001). Significant reductions in salivary MMP‐8 concentrations were detected by the biosensor (*p* = .030) and IFMA (*p* = .002) in participants with periodontitis 6 months after non‐surgical periodontal treatment. IFMA had the best sensitivity (89.2%) for detecting periodontitis and gingivitis versus health and 96.6% for detecting periodontitis versus health and gingivitis. The biosensor had an AUC value of 0.81 and diagnostic accuracy of 74.2% for differentiating periodontitis and gingivitis from health; an AUC value of 0.86 and diagnostic accuracy of 82.8% for periodontitis versus health and gingivitis.

**Conclusions:**

The biosensor, IFMA, and ELISA assays differentiated between periodontal health, gingivitis, and periodontitis based on salivary MMP‐8 levels. Only the biosensor and, particularly, IFMA identified an effect of periodontal treatment in the participants with periodontitis. Our findings support the potential utility of salivary oral fluid aMMP‐8‐based point‐of‐care technology in the future of periodontal diagnostics.

## 1 INTRODUCTION

Periodontitis is a chronic inflammatory disease that results in progressive destruction of the periodontal tissues and remains a significant cause of tooth loss in adults, with concomitant negative impacts on oral health‐related quality of life.^1^ Severe periodontitis is the sixth‐most prevalent disease globally, with consequent adverse effects on oral health as well as contributing to systemic inflammation.^2^, ^3^, ^4^ Periodontitis poses a huge health and economic burden globally.^2^, ^4^ Early diagnosis of periodontitis is therefore a key strategy to facilitate timely and more effective interventions and to achieve a better long‐term prognosis.^5^

Periodontitis is diagnosed through clinical and radiographic examination.^6^, ^7^ However, it is recognized that these traditional diagnostic methods have some shortcomings: often they reflect past disease activity and can be time‐consuming and technically challenging to undertake, as well as being somewhat subjective, being dependent on the expertise and proficiency of the clinician. There is, therefore, potential benefit in developing additional diagnostic methods that can objectively assess current and future periodontal disease activity.

The potential usefulness of disease‐specific inflammatory biomarkers such as matrix metalloproteinase (MMP)‐8 in oral fluids (saliva, gingival crevicular fluid (GCF), peri‐implant sulcular fluid (PISF), and mouth rinses) has been demonstrated in several studies that have correlated MMP‐8 with periodontal disease course and severity.^8^, ^9^, ^10^, ^11^, ^12^ MMP‐8, otherwise known as neutrophil collagenase or collagenase‐2, is the major collagenolytic enzyme released by neutrophils and is a key mediator in most of the connective tissue destruction in inflammatory periodontal disease and peri‐implantitis.^11^, ^13^, ^14^ Recently, the potential utility of the active form of MMP‐8 (aMMP‐8), as a biomarker in the oral‐systemic link was highlighted, due to the contribution of periodontitis to the inflammatory burden in various systemic diseases and conditions.^15^ Active MMP‐8, but not the total or latent form, is related to and predicts the progression of periodontitis due to its catalytic activity in oral fluids.^10^, ^16^, ^17^, ^18^, ^19^, ^20^, ^21^

Antibody‐based immunoassays utilizing monoclonal antibodies such as the standard laboratory time‐resolved immunofluorometric assay (IFMA) and enzyme‐linked immunosorbent assay (ELISA) can detect MMP‐8 in oral fluids.^22^, ^23^ IFMA correlates more strongly with periodontal and peri‐implant tissue destruction than commercially available ELISA kits, which frequently detect total MMP‐8 and cannot readily distinguish between different MMP‐8 forms in periodontal health and disease.^10^, ^13^, ^19^, ^22^, ^24^, ^25^, ^26^, ^27^ Assays that measure biomarkers could be useful in monitoring the progression of periodontal disease and the response to treatment.^11^, ^28^, ^29^, ^30^ Some of these assays can facilitate the rapid detection of aMMP‐8 enzymatic levels in 5–7 minutes, thus offering potential for early diagnosis of periodontal disease (PerioSafe^®^, ORALyzer^®^).^31^, ^32^ The relevance of biomarkers has been highlighted in the staging and grading system for the classification of periodontitis, as potentially improving diagnostic accuracy.^1^ In line with this, it was proposed that aMMP‐8 could be the oral biomarker of choice to be used in the staging and grading of periodontitis.^31^, ^33^, ^34^

Laboratory‐based IFMA, ELISA, and chairside lateral flow immunoassays are currently the most widely available methods of quantifying MMP‐8 in oral fluids.^34^, ^35^, ^36^, ^37^ Recently, MMP‐8 enzymatic activity was detected and quantified in the GCF of periodontally diseased sites and found to be significantly higher than healthy sites.^20^ Also, molecular forms of neutrophilic and mesenchymal‐type MMP‐8 such as 20–27 kDa fragments were shown to be elevated in periodontitis, suggesting a potential role as early diagnostic markers of active periodontal disease.^38^ However, these are still at early experimental stages, and the variability in the specificity and sensitivity of the available assays has stimulated the search for other oral fluid point‐of‐care diagnostic methodologies that have greater precision. In this regard, recently, a novel prototype biosensor was developed and utilized to quantify salivary MMP‐8 using specific antibodies and surface acoustic wave (SAW) technology in patients with periodontal disease.^5^ Accordingly, in the present study, we aimed to compare the diagnostic utility of the SAW biosensor with other antibody‐based assays (IFMA to measure aMMP‐8 and ELISA to measure total MMP‐8 [tMMP‐8]) in subjects with periodontal health, gingivitis, and periodontitis before and after treatment.

## 2 MATERIAL AND METHODS

### 2.1 Study design and setting

The clinical phase of this cohort study was conducted at the Dental Clinical Research Facility of Newcastle Dental Hospital, Newcastle upon Tyne NHS Foundation Trust, UK, from 2012 to 2016. All subjects provided written informed consent, and the ethical approval was received from the National Research Ethics Service North East Newcastle and North Tyneside 1 committee (Ref: 12/NE/0396). SAW analyses were undertaken at Newcastle University, whereas IFMA and ELISA analyses were undertaken at the University of Helsinki.

### 2.2 Subjects/patients and clinical assessments

Details of the clinical study have been previously published.^5^ Briefly, samples from 189 participants were assessed in this study. The inclusion criteria were adults ≥18 years of age, systemically healthy, non‐smokers, with a minimum of 20 natural teeth excluding third molars. Exclusion criteria included periodontal treatment within 6 months prior to the study, removable partial dentures or orthodontic appliances, xerostomia, the use of medications that could affect the periodontal tissues and current use of antibiotics, immunosuppressants, or non‐steroidal anti‐inflammatory drugs. The periodontal parameters assessed included clinical attachment loss (CAL), periodontal probing depth (PPD), modified gingival index (mGI),^39^ and percentage bleeding on probing (%BOP), recorded using a manual periodontal probe (UNC‐15, Hu‐Friedy) at six sites per tooth. The patients were allocated into three groups: healthy participants had PPD of ≤3 mm at all sites, no sites with interproximal attachment loss, mGI ≥2.0 in ≤10% of sites and BOP ≤10%; gingivitis patients had mGI of ≥3.0 in ≥30% of sites, no sites with interproximal attachment loss or PPD ≥4 mm, and BOP ≥10%; periodontitis patients had interproximal PPD ≥5 mm at ≥8 teeth and BOP ≥30%.

Participants with periodontitis received non‐surgical periodontal treatment (oral hygiene instruction, root surface debridement using a combination of manual and ultrasonic instruments under local anesthesia) and were re‐assessed after 6 months.

### 2.3 Saliva sample collection

Unstimulated saliva samples (3–5 ml) were collected by expectoration into sterile plastic centrifuge tubes. The samples were immediately placed on ice after collection and centrifuged for 15 minutes at 1500 g and 4°C. 500 µl aliquots were taken, then frozen in liquid nitrogen, and stored at −80°C until analysis. The saliva samples were collected at baseline from all participants and from twenty (*n* = 20) periodontitis patients at 6 months following non‐surgical periodontal therapy.

### 2.4 Salivary MMP‐8 biosensor

The biosensor technology had been described in previous studies.^5^, ^40^, ^41^ In summary, it comprises a disposable Surface Acoustic Wave (SAW) biochip coated with specific antibodies that deliver a signal to a control box upon detection of analyte (MMP‐8). Subsequently, this signal is converted into a digital representation, which is processed by a designated software received by a laptop PC. The biochip has interdigitating input and output gold electrodes connected by a gold‐film‐coated sensing area built on a piezoelectric quartz crystal. This formation enables the excitation of a shear horizontal SAW of specific wavelength and frequency. Capture antibodies on the gold‐film surface thus becomes sensitive to antigen binding by means of amplitude and velocity changes in the SAW signal. Thus, MMP‐8 antigen in the sample binds to the antibodies and the perturbation caused by the antigen/antibody binding is detected by the difference in wave phases between the input and output electrodes (i.e., phase change, Δϕ). The novel prototype device has an assay time of 20 minutes and detection limit of 62.5 ng/ml.^5^

### 2.5 Salivary aMMP‐8 Immunofluorometric assay

A time‐resolved immunofluorometric assay (IFMA) was used to assess aMMP‐8 concentrations in saliva based on the original description.^42^ Summarily, the monoclonal MMP‐8‐specific antibodies 8708 and 8706 (Medix Biochemica) were used as catching and tracer antibodies, respectively. The tracer antibody was then labeled using europium‐chelate. The assay buffer contained 20 mM Tris‐HCl (pH 7.5), 0.5 M NaCl, 5 mM CaCl_2_, 50 mM ZnCl_2_, 0.5% bovine serum albumin, 0.05% sodium azide, and 20 mg/L diethylenetriaminepentaacetic acid (DTPA). The saliva samples were diluted in assay buffer and incubated with the capture antibody for 1 hour, followed by incubation with the tracer antibody for 1 hour. Enhancement solution was added, and after 5 minutes, fluorescence was measured using EnVision 2105 Multimode Plate Reader (PerkinElmer Finland). The specificity of the monoclonal antibodies against aMMP‐8 corresponded to that of the polyclonal MMP‐8 and the detection limit for the assay is 0.08 ng/ml.

### 2.6 Salivary total/latent MMP‐8 ELISA

The MMP‐8 concentration in the saliva samples was detected using a commercially available ELISA (Quantikine Human Total MMP‐8 Immunoassay R&D Systems™) according to the manufacturer's instructions and established protocols.^35^, ^43^, ^44^ Duplicate salivary samples were assessed for the enzyme levels in each participant. ELISA has a detection limit of 0.06 ng/ml, and all data points were within the linear range of the assay.

### 2.7 Statistical analysis

The statistical analyses were carried out with SPSS version 25.0. Differences in continuous variables such as aMMP‐8/total MMP‐8 levels between the three groups were assessed using Kruskal–Wallis tests. In addition, post hoc tests were performed with Dunn–Bonferroni post hoc method while the Spearman's rank correlation was used to determine the correlations between Biosensor, ELISA (total MMP‐8), aMMP‐8 IFMA, and the clinical indices. Wilcoxon signed‐rank test was used to compare differences in salivary MMP‐8 concentrations pre‐ and post‐non‐surgical periodontal treatment. Diagnostic accuracy of MMP‐8 analysis methods for the biosensor, IFMA and ELISA were determined using receiver operating characteristic (ROC) curve analysis. The level of statistical significance was set at *p* < .05.

## 3 RESULTS

### 3.1 Demographic characteristics

Following clinical examination, 59 participants were included in the healthy group, 63 participants were identified as having gingivitis, and 67 participants had periodontitis. Females (*n* = 118) represented 62.4% of the total study population (*n* = 189), while the mean age of all participants was 40.4 ± 11.7 years (range 18–62 years).

### 3.2 Correlation of MMP‐8 of biosensor (phase change, Δφ) with aMMP‐8 IFMA and ELISA

Data related to the salivary measurement of MMP‐8 using the SAW biosensor have been previously published.^5^ In the present study, salivary MMP‐8 levels determined by the SAW biosensor had a significant correlation with aMMP‐8 IFMA (ng/ml; *r* = .354, *p* < .001) and total MMP‐8 ELISA (ng/ml; *r* = .681, *p* = .001) among all the participants (Table 1). With respect to the healthy volunteers, salivary MMP‐8 as measured by the biosensor showed a significant correlation with total MMP‐8 as measured by ELISA (ng/ml; *r* = .436, *p* = .002) but not with the measurements from the aMMP‐8 IFMA (Table 1). Among the gingivitis patients, MMP‐8 levels measured using the SAW biosensor had no significant correlation with either the aMMP‐8 IFMA or total MMP‐8 ELISA (Table 1). However, in the periodontitis group, MMP‐8 assayed using the SAW biosensor significantly correlated with total MMP‐8 measured by ELISA (ng/ml; *r* = .450, *p* < .001) but not with aMMP‐8 measured by IFMA (ng/ml). Table 2 shows the correlations between aMMP‐8 IFMA and MMP‐8 ELISA. Overall, this correlation was significant and strong (*r* = .608, *p* < .001). In periodontal health, the correlation was also significant and strong (*r* = .700, *p* < .001) and for gingivitis (*r* = .482, *p* < .001) but was not significant for periodontitis.

**TABLE 1:** Correlation of salivary MMP‐8 biosensor (Δφ) with IFMA and ELISA assays in study population

MMP‐8 biosensor (Δφ) vs.	aMMP‐8 IFMA (ng/ml)		MMP‐8 ELISA (ng/ml)	
Periodontal status	Spearman's rho	*p*‐value	Spearman's rho	*p*‐value
Healthy (*n* = 59)	0.252	.081	0.436	.002*
Gingivitis (*n* = 63)	0.138	.370	0.247	.106
Periodontitis (*n* = 67)	−0.068	.612	0.450	<.001*
Total (*n* = 189)	0.354	<.001*	0.681	<.001*

* Significant; Spearman's rho (rank correlation test)

**TABLE 2:** Correlation of salivary aMMP‐8 IFMA (ng/ml) with MMP‐8 ELISA

aMMP‐8 IFMA vs.	MMP‐8 ELISA (ng/ml)	
Periodontal status	Spearman's rho	*p*‐value
Healthy (*n* = 59)	0.700	<.001*
Gingivitis (*n* = 63)	0.482	<.001*
Periodontitis (*n* = 67)	0.177	.158
Total (*n* = 189)	0.608	<.001*

* Significant; Spearman's rho (rank correlation test).

### 3.3 Comparative analysis of the performance of salivary MMP‐8 assay methods

**FIGURE 1:**
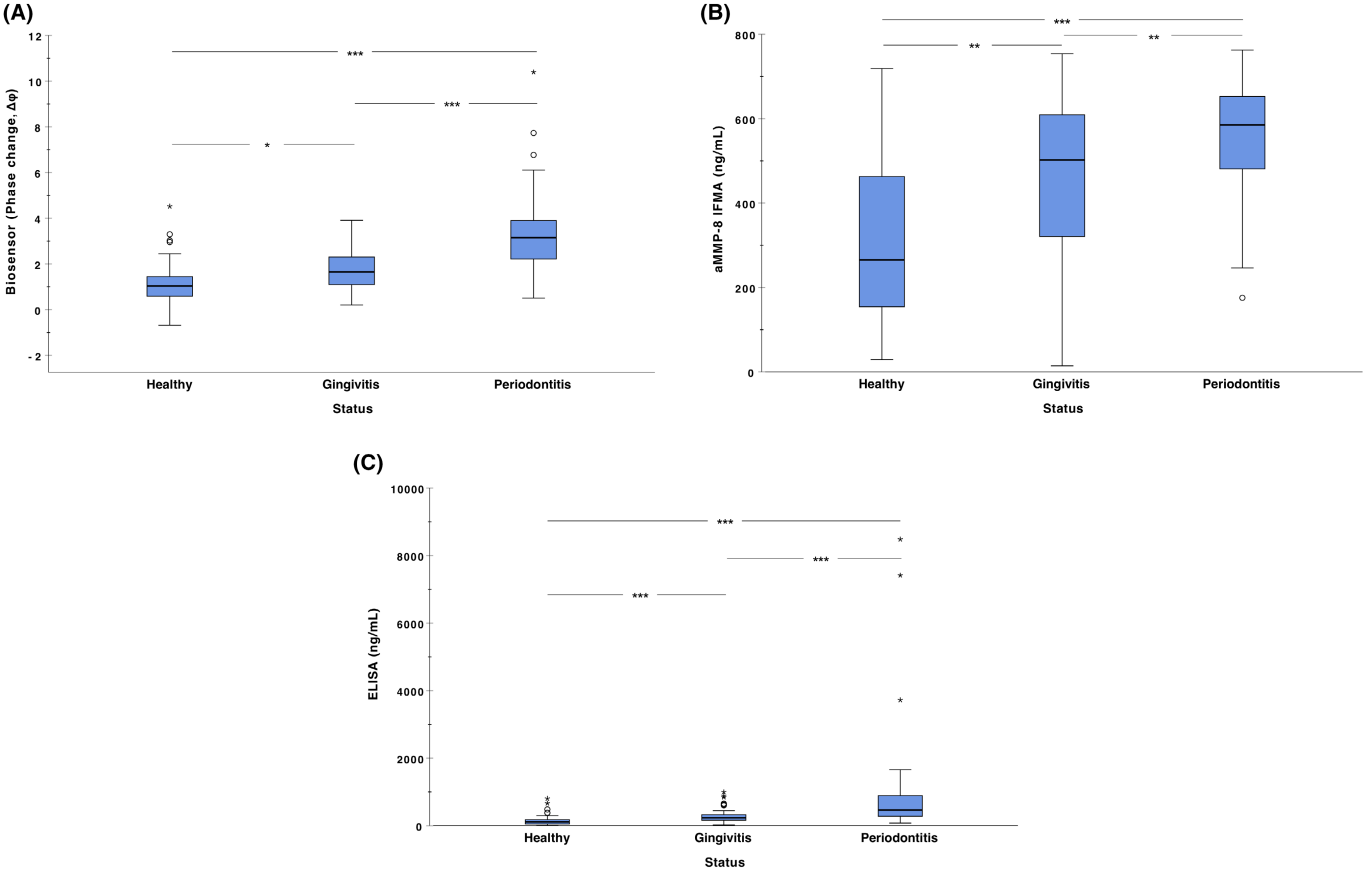
Differences in salivary MMP‐8 levels between the healthy (*n* = 59), gingivitis (*n* = 63), and periodontitis (*n* = 67) participants based on (A) biosensor (B) aMMP‐8 IFMA and (C) ELISA. The data are shown as box and whisker plots and analyzed using Kruskal–Wallis tests, while the post hoc tests were performed with Dunn–Bonferroni post hoc method. *p < .05, **p < .01, ***p < .001

The comparative performance of the biosensor and the other assay methods for salivary MMP‐8 in terms of distinguishing periodontal health, gingivitis, and periodontitis was assessed. (Table 3) There were significant differences in the salivary MMP‐8 levels between healthy and gingivitis participants as measured by the biosensor (*p* < .05, Figure 1A), aMMP‐8 IFMA (*p* < .001, Figure 1B), and total MMP‐8 ELISA (*p* < .001, Figure 1C). Salivary MMP‐8 levels were also significantly different between healthy and periodontitis subjects as assayed by the biosensor (*p* < .001, Figure 1A), aMMP‐8 IFMA (*p* < .001, Figure 1B), and total MMP‐8 ELISA (*p* < .001, Figure 1C) and significantly different between gingivitis and periodontitis participants as measured by the biosensor (*p* < .001, Figure 1A), aMMP‐8 IFMA (*p* < .01, Figure 1B) and total MMP‐8 ELISA (*p* < .001, Figure 1C).

**TABLE 3:** Comparison of changes in mean rank of salivary MMP‐8 levels in study population

	Change in mean rank Biosensor (Δφ)	*p*‐value	Change in mean rank aMMP‐8 IFMA (ng/ml)	*p*‐value	Change in mean rank MMP‐8 ELISA (ng/ml)	*p*‐value
Healthy vs Gingivitis	22.77	.037*	34.99	.001*	40.01	<.001*
Gingivitis vs Periodontitis	41.84	<.001*	29.56	.006*	45.78	<.001*
Healthy vs Periodontitis vs	64.60	<.001*	64.54	<.001*	85.78	<.001*

* Significant for pairwise post hoc analysis for mean rank of periodontal status.

### 3.4 ROC‐curve analysis

**FIGURE 2:**
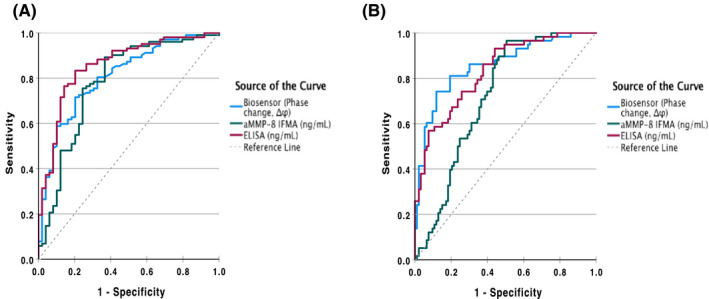
ROC curves of MMP‐8 analysis methods Biosensor, IFMA, and ELISA in classifying between (A) periodontitis and gingivitis versus health; and (B) periodontitis versus health and gingivitis

The receiver operating characteristic (ROC) analysis and diagnostic performance of the salivary MMP‐8 assay methods in differentiating between periodontitis, gingivitis, and health, depicted by the area under the curve (AUC) values are shown on Table 4, Figure 2A,B. The biosensor, IFMA, and ELISA assays had high AUC values of 0.808, 0.782, and 0.857, respectively, for differentiating periodontitis and gingivitis versus health. In discriminating periodontitis versus health and gingivitis, the AUC values for biosensor, IFMA and ELISA were 0.857, 0.720, and 0.832, respectively (Table 4). The sensitivities of the biosensor, IFMA, and ELISA assays were 71.6%, 89.2%, and 83.3%, respectively, for periodontitis and gingivitis versus health, while for periodontitis versus health and gingivitis, they were 74.1%, 96.6%, and 56.9%, respectively.

**TABLE 4:** ROC analysis and diagnostic performance of MMP‐8 analysis methods Biosensor, IFMA and ELISA in classifying between (A) periodontitis and gingivitis versus health; and (B) periodontitis versus health and gingivitis

Periodontitis + Gingivitis vs Health									
	AUC	Cutoff	OR	Se(%)	Sp(%)	FN(%)	FP(%)	Acc(%)	MCC
Biosensor	0.808 (0.736–0.881)	1.6	9.8	71.6	79.6	42.6	12.0	74.2	0.48
IFMA	0.782 (0.696–0.867)	347.7	14.2	89.2	63.3	26.2	16.5	80.8	0.55
ELISA	0.857 (0.792–0.922)	187.6	19.5	83.3	79.6	30.4	10.5	82.1	0.61

Periodontitis vs Health + Gingivitis									
	AUC	Cutoff	OR	Se(%)	Sp(%)	FN(%)	FP(%)	Acc(%)	MCC
Biosensor	0.857 (0.793–0.920)	2.5	21.4	74.1	88.2	15.5	20.4	82.8	0.63
IFMA	0.720 (0.641–0.800)	378.3	27.4	96.6	49.5	4.2	45.6	67.5	0.48
ELISA	0.832 (0.768–0.896)	386.5	16.2	56.9	92.5	22.5	17.5	78.8	0.54

Note

Cutoff calculated by Youden's index.

Abbreviations: Acc, accuracy; FN, false negatives; FP, false positives; MCC, Matthews correlation coefficient; OR, odds ratio; Se, sensitivity; Sp, specificity.

### 3.5 Analysis of salivary MMP‐8 before and 6 months after non‐surgical periodontal therapy

**FIGURE 3:**
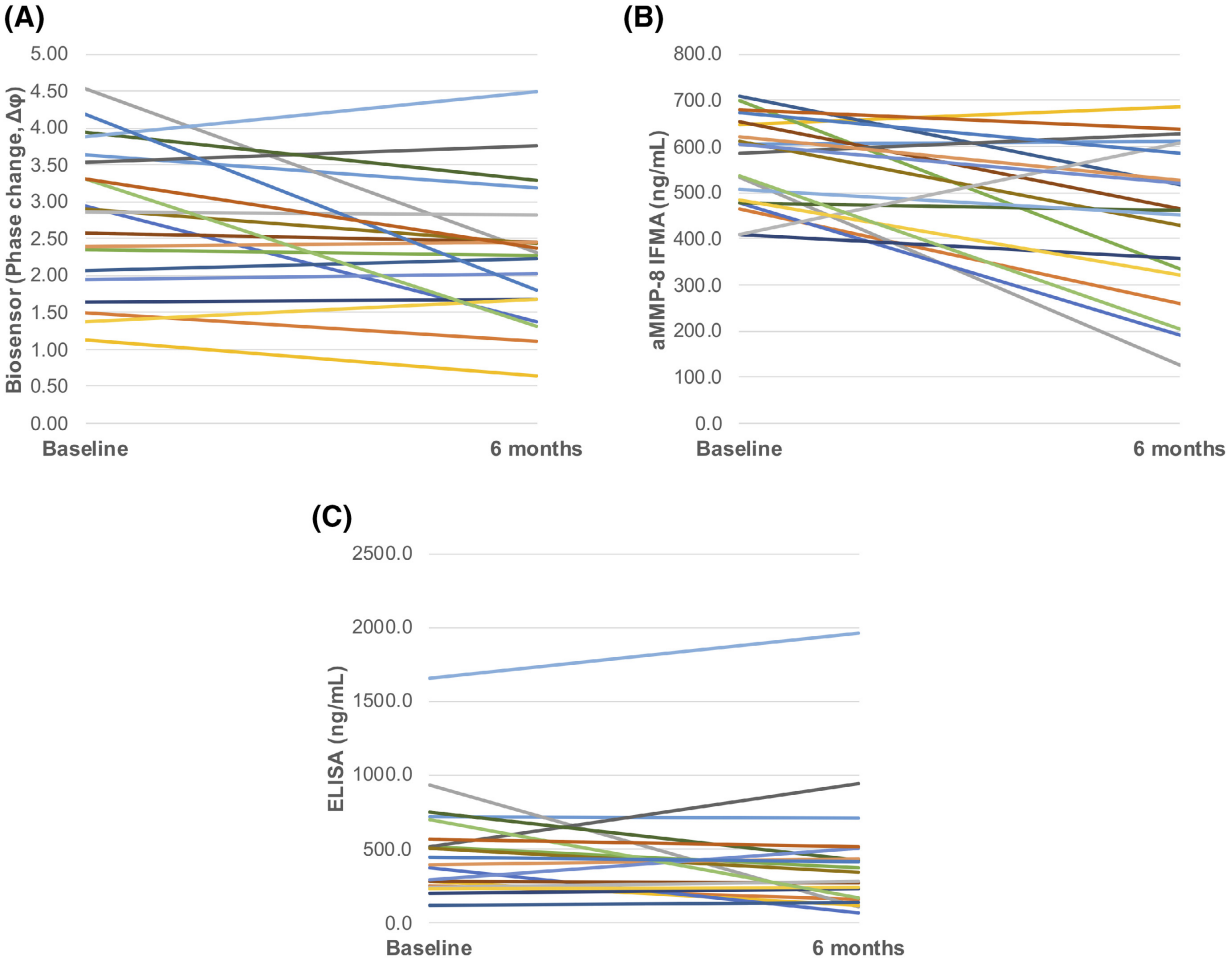
Differences in salivary MMP‐8 levels in periodontitis patients (*n* = 20) at baseline and 6 months after non‐surgical periodontal treatment. (A) Biosensor (*p* < .05; B) aMMP‐8 IFMA (*p* < .01) and (C) ELISA (*p* > .05)

The ability of the assays to detect changes in salivary MMP‐8 levels before and 6 months after non‐surgical periodontal treatment was also evaluated in a sub‐set of twenty periodontitis patients (Table 5). The biosensor assay showed a significant reduction in salivary MMP‐8 levels (*p* = .016; Figure 3A) 6 months after periodontal treatment. This reduction was more significant in aMMP‐8 IFMA after 6 months (*p* = .002; Figure 3B) but the pre‐ and post‐treatment levels of MMP‐8 as measured by the total MMP‐8 ELISA were not significantly different (*p* = .221; Figure 3C). It is also important to note that only six and four subjects had persistently elevated MMP‐8 levels as measured by the biosensor and the IFMA assay, respectively.

**TABLE 5:** Comparative analysis of mean salivary MMP‐8 levels in periodontitis patients (*n* = 20) at baseline and 6 months after periodontal treatment (*n* = 20)

Assay method	Mean MMP‐8 levels		Mean difference	*p*‐value
	Baseline (t0)	6 months (t6)		
Biosensor (Δφ)	2.80 ± 0.99	2.29 ± 0.92	0.52 ± 0.87	.030*
aMMP‐8 IFMA (ng/ml)	569.54 ± 95.41	445.92 ± 165.07	123.62 ± 151.94	.002*
ELISA (ng/ml)	496.91 ± 347.78	418.45 ± 422.63	78.46 ± 277.52	.232

* Significant by Wilcoxon signed‐rank test.

## 4 DISCUSSION

In this study, we compared the potential utility of a novel biosensor for detecting MMP‐8 in saliva samples with an established laboratory IFMA which is selective for aMMP‐8, and a commercial ELISA immunoassay which is selective for total/latent MMP‐8. The biosensor utilizes specific antibodies coated on a mini biochip to quantify total MMP‐8 (inactive total/latent pro‐MMP‐8) through a microelectromechanical piezoelectric SAW technology which generates a digital readout to reflect the salivary MMP‐8 levels.^5^

The significantly higher salivary MMP‐8 levels detected in gingivitis and periodontitis participants compared with healthy participants by all the three assays, aligns with previous studies that have utilized IFMA, ELISA and quite recently the biosensor.^5^, ^11^, ^19^, ^24^, ^33^, ^35^, ^36^, ^45^, ^46^ Recently, the ability of aMMP‐8 IFMA and PerioSafe^®^ point‐of‐care/chairside assay to distinguish different stages of periodontal disease (gingivitis, periodontitis stages III and IV) from periodontal health was demonstrated using both GCF and saliva.^19^ In another study, the aMMP‐8 IFMA assay also differentiated between patients with periodontal health, gingivitis, and periodontitis stage III, grade C based on higher salivary aMMP‐8 concentrations.^33^ Higher aMMP‐8 catalytic activity has also been demonstrated in the GCF and saliva of periodontally diseased sites and patients compared with healthy sites and patients.^20^, ^47^, ^48^

The elevated aMMP‐8 levels observed in periodontitis result in active enzymatic degradation of interstitial type I collagen fibers of the periodontal tissues.^24^, ^48^ The majority (90%–95%) of the collagenolytic activity in GCF originates from aMMP‐8, and its elevation associates closely with disease severity.^11^, ^13^, ^24^, ^32^, ^33^, ^37^, ^48^, ^49^ In addition, it has been highlighted that the activated form of MMP‐8 released by the polymorphonuclear neutrophils (PMNs), and not the total or latent form of MMP‐8, accurately detects and predicts periodontal tissue destruction.^21^, ^24^, ^28^, ^32^, ^50^, ^51^, ^52^ The fact that in the post hoc analyses, the assays clearly demarcated MMP‐8 levels between health and gingivitis, health and periodontitis, and gingivitis and periodontitis is relevant. Potentially, they could be used to facilitate the timing of targeted and personalized treatment.

In this study, we have confirmed the correlation of the salivary MMP‐8 biosensor with MMP‐8 ELISA data.^5^ We also report for the first time a correlation between the levels of salivary tMMP‐8 as measured by the biosensor and aMMP‐8 as measured by IFMA, suggesting the SAW biosensor system is capturing quantitatively the active MMP‐8 fraction of the total MMP‐8 in saliva.

The differences in correlations between the biosensor and the other salivary MMP‐8 assays in comparison between healthy, gingivitis, and periodontitis groups in the current study could be partly attributed to different specificities and sensitivities between the antibodies used in the three assays. The affinity of the specific antibodies to detect the active and latent forms of MMP‐8 differs. The biosensor had a stronger correlation for ELISA in the current study, as both assay methods detect total MMP‐8 (active + latent), as ELISA cannot differentiate the active from latent forms of MMP‐8.^16^, ^21^, ^53^ (Gul et al., 2020) In contrast, there was a weak correlation between the biosensor and IFMA in healthy and periodontitis groups. IFMA has a high affinity for the active MMP‐8 which is the molecular form associated with the onset and progression of periodontitis.^10^, ^11^, ^22^

The difference in results from the three MMP‐8 assays following periodontal treatment has been demonstrated previously.^32^, ^54^ In the present study, measurements using both the IFMA and biosensor assays demonstrated significant differences in salivary MMP‐8 levels in periodontitis patients before, and 6 months after, treatment, although the statistical significance was greater with the IFMA assay. The ability of IFMA to detect a treatment effect in aMMP‐8 levels following periodontal therapy confirms previous findings.^10^ IFMA detects mainly aMMP‐8 which is predominant in oral fluids in periodontitis.^10^, ^11^, ^16^ The SAW biosensor, however, detects total MMP‐8 which can also be assessed by the ELISA.^5^ The significant reduction in aMMP‐8 post‐treatment detected by the IFMA further corroborates the key role of MMP‐8 in the pathogenesis of periodontitis.^11^, ^24^, ^32^, ^37^

The SAW biosensor data correlated more strongly with MMP‐8 ELISA data but less so with aMMP‐8 IFMA possibly because most of the MMP‐8 in saliva is in the total and latent form.^20^, ^28^, ^48^, ^51^, ^55^, ^56^ The significant correlation between salivary MMP‐8 levels measured by the biosensor and ELISA has also been reported.^5^ This can also be explained, at least in part, by the fact that the biosensor utilized MMP‐8 antibody that is selective for total MMP‐8 (similar to the ELISA) while IFMA utilized the aMMP‐8 selective antibody.^57^

As previously documented, aMMP‐8 measured by IFMA correlates more strongly with periodontitis sites and has a higher diagnostic accuracy than ELISA.^10^, ^11^, ^22^, ^57^, ^58^ The diagnostic performance of the assay methods to discriminate periodontitis, gingivitis, and health was further determined by a ROC‐curve analysis. IFMA had the best sensitivity (89.2%) for detecting periodontitis and gingivitis versus health, and 96.6% for detecting periodontitis versus health and gingivitis. The AUC value of IFMA in discriminating periodontitis and gingivitis from health was 0.78 and less than those of the biosensor (0.81) and ELISA assays (0.86). In addition, the biosensor and ELISA assays had very good diagnostic AUC values of 0.86 and 0.83, respectively, for periodontitis versus health and gingivitis, which were higher than the IFMA assay (0.72).

The diagnostic performance of the SAW biosensor has been reported previously,^5^ and the present study further supports its potential utility in future periodontal diagnostics. We recognize that other oral diseases and conditions, such as caries activity and reduced salivary flow rate, could affect MMP‐8 assays, potentially through the activation of pro‐MMP‐8 by acids produced by cariogenic bacteria.^59^These factors will need to be considered when future studies on the utility of salivary MMP‐8 assays are conducted.

## 5 CONCLUSION

In summary, this study has reaffirmed the ability of the SAW biosensor, IFMA, and ELISA assays to detect MMP‐8 levels in saliva to distinguish participants with periodontal health, gingivitis, and periodontitis. Both the biosensor and the IFMA (aMMP‐8) detected a periodontal treatment effect among the periodontitis participant, as indicated by the reduced salivary MMP‐8 levels after 6 months. The diagnostic utility of the biosensor and ELISA assays was demonstrated in differentiating between periodontal health, gingivitis, and periodontitis. Overall, the study findings strongly indicate the potential usefulness of, in particular, the oral fluid aMMP‐8‐based technologies for point‐of‐care periodontal assessment in the future.

## CONFLICT OF INTEREST

Prof Timo Sorsa is the inventor of U.S. patents 5652223, 5736341, 5864632, 6143476 and US 2017/0023571A1 (issued June 6, 2019), WO 2018/060553A1 (issued May 31, 2018), and 10488415B2, Japanese Patent 2016‐554676 and South Korean patent 10‐2016‐70225378. Other authors declare no conflicts of interest. The funders had no role in the design of the study; in the collection, analyses, or interpretation of data; in the writing of the manuscript, or in the decision to publish the results.

## AUTHOR CONTRIBUTIONS

TS, JJT, PP, PMP, ITR, SMB, KAU and SON contributed to the study conception and research design; PP, TT, HL and SMB performed the data collection for the laboratory and clinical aspects; KAU, TT, PMP, JJT, ITR and TS analyzed the data; TS, PP, JJT, SMB, KAU, SON and HL interpreted the data; KAU, PP, PMP, ITR, HL, SMB, SON and TS drafted the manuscript; KAU, PP, PMP, JJT, ITR, SMB, SON and TS revised the manuscript for initial intellectual content; and all the authors read and approved the final manuscript.

## Data Availability

The data that support the findings of this study are available from the corresponding author upon reasonable request.
